# Supplementary material for: Plant Extracts from the Yucatan Peninsula in the In Vitro Control of Curvularia lunata and Antifungal Effect of Mosannona depressa and Piper neesianum Extracts on Postharvest Fruits of Habanero Pepper
Source: Plants (Basel). 2023 Aug 9;12(16):2908. doi: 10.3390/plants12162908 (PMC10459550; doi:10.3390/plants12162908)
Supplement: Supplementary file 1 [file plants-12-02908-s001.zip › plants-2499562-supplementary.pdf]

Table S1. Inhibition of mycelial growth of *Curvularia lunata* strain (ITC26) by plant extracts from 40 native species of the Yucatán Peninsula in microdilution assay

	Mycelial Growth Inhibition (%)							
	Ethanolic extract (2,000 μg/mL)				Aqueous extract (3%, p/v)			
**Plant species**	**L**	**S**	**R**	**WP**	**L**	**S**	**R**	**WP**
Alseis yucatanensis	0	ne	ne	ne	0	ne	ne	ne
Alvaradoa amorphoides	75	0	0	ne	0	0	0	ne
Annona primigenia	0	0	ne	ne	0	0	ne	ne
Bakeridesia notolophium	0	0	ne	ne	0	0	ne	ne
Bravaisia berlandieriana	0	0	0	ne	0	0	0	ne
Byrsonima bucidifolia	0	0	0	ne	25	25	25	ne
Calea jamaicensis	ne	ne	ne	0	ne	ne	ne	0
Cameraria latifolia	0	0	0	0	0	0	0	0
Chrysophyllum mexicanum	0	0	0	0	0	0	0	ne
Coccoloba sp.	0	0	0	ne	0	0	0	ne
Croton arboreus	0	0	0	0	0	0	0	0
Croton itzaeus	0	0	0	0	0	0	0	0
Croton sp.	ne	ne	ne	0	ne	ne	ne	0
Cupania sp.	0	0	ne	ne	0	0	ne	ne
Diospyros sp.	0	ne	ne	ne	0	ne	ne	ne
Erythroxylum confusum	0	0	0	0	0	0	0	0
Erythroxylum rotundifolium	0	0	ne	ne	0	0	ne	ne
Erythroxylum sp.	0	ne	ne	ne	0	ne	ne	ne
Eugenia sp.	0	0	0	ne	0	0	0	ne
Euphorbia armourii	ne	ne	ne	0	ne	ne	ne	0
Guettarda combsii	0	0	0	ne	0	0	0	ne
Helicteres baruensis	75	25	0	ne	0	0	0	ne

Heteropterys laurifolia	0	0	0	ne		0	0	0	ne	
Hybanthus yucatanensis	0	0	ne	ne		0	0	ne	ne	
Ipomoea clavata	ne	ne	ne	0		ne	ne	ne	0	
Karwinskia humboldtiana	0	ne	ne	ne		0	ne	ne	ne	
Licaria sp.	0	0	75	ne		0	0	0	ne	
Macroscepis diademata	0	0	ne	ne		0	0	ne	ne	
Malpighia glabra	0	0	0	ne		0	0	0	ne	
Morella cerifera	0	0	0	ne		25	0	25	ne	
Mosannona depressa	0	100	100	ne		0	0	0	ne	
Parathesis cubana	0	0	0	ne		0	0	0	ne	
Paullinia sp.	0	ne	0	ne		0	ne	0	ne	
Piper neesianum	100	0	75	ne		0	0	0	ne	
Psychotria nervosa	0	0	0	ne		0	0	0	ne	
Randia aculeata	0	0	0	ne		0	0	0	ne	
Serjania caracasana	0	0	0	ne		0	0	0	ne	
Simarouba glauca	0	0	0	ne		0	0	0	ne	
Stemmadenia donnell-smithii	0	0	ne	ne		0	0	ne	ne	
Turnera aromatica	ne	ne	ne	0		ne	ne	ne	0	
RPMI					0					0
blank					0					0
Prochloraz 0.11%					100					100

C: control; L: leaves; S: stem, R: root; WP: whole plant; ne: not evaluated; RPMI: Roswell Park Memorial Institute medium; blank: dimethyl sulfoxide with 0.5% Tween 20. Extracts from *M. depressa* were from barks of stems and roots.
